# Does Advanced Imaging Aid in the Preoperative Evaluation of Patients With Moyamoya Disease?

**DOI:** 10.7759/cureus.29816

**Published:** 2022-10-01

**Authors:** Tim White, Shashank Gandhi, David J Langer, Jeffrey M Katz, Amir R Dehdashti

**Affiliations:** 1 Neurosurgery, Hofstra Northwell School of Medicine, Manhasset, USA; 2 Neurosurgery, Barrow Neurological Institute, Phoenix, USA; 3 Neurosurgery, Brain Tumor Center, Hofstra Northwell School of Medicine, Manhattan, USA; 4 Neurology, Hofstra Northwell School of Medicine, Manhasset, USA; 5 Neurological Surgery, Northwell Health, Manhasset, USA

**Keywords:** moyamoya disease, quantitative magnetic resonance angiography, encephaloduroarteriosynangiosis (edas), cerebral revascularization, sta-mca bypass, moyamoya disease (mmd)

## Abstract

Background

Moyamoya disease is characterized by progressive nonatherosclerotic stenosis and eventual occlusion of the supraclinoid cerebral arteries with the associated development of abnormal collateral vessels. Treatment of moyamoya disease revolves around restoring cerebral blood flow (CBF) distal to the steno-occlusive disease. Numerous modalities can be used to assess hemodynamic parameters. We sought to determine the impact of preoperative imaging on surgical decision-making.

Methods

A retrospective review was performed of all patients seen with the diagnosis of moyamoya. Patients were grouped on presentation based on CT/MRI findings of infarction, hemorrhage, or normal. Patients who did not have all of the preoperative tests were excluded. Preoperative radiological results were dichotomized as either normal or abnormal.

Results

During a five-year period, 34 patients with moyamoya met the inclusion criteria. All patients had an abnormal magnetic resonance angiography (MRA) Non-invasive Optimal Vessel Analysis (NOVA; VasSol, Inc, River Forest, IL). Three patients had normal initial MRI. All symptomatic patients had abnormal preoperative workup and underwent revascularization, as all were found to have abnormal single photon emission computed tomography (SPECT). The only occasion where the decision for surgery or type of surgery was influenced by imaging findings was in patients with nonclassical or minimal symptoms.

Conclusion

Although hemodynamic imaging studies can aid in establishing a preoperative baseline of CBF and cerebral vascular reserve (CVR) for follow-up studies, the true implication of these tests in the preoperative evaluation of clearly symptomatic moyamoya patients is debatable. In asymptomatic/mildly symptomatic patients, hemodynamic studies are necessary to determine the need for treatment. For symptomatic patients, surgery can be performed without an exhaustive and costly preoperative hemodynamic evaluation.

## Introduction

Moyamoya disease is characterized by progressive nonatherosclerotic stenosis and eventual occlusion of the supraclinoid cerebral arteries with the associated development of abnormal collateral vessels [1,2]. The gradual occlusion of these vessels leads to cerebral perfusion pressure reductions and decreased cerebral blood flow (CBF) [3]. The capacity of the vessels to compensate for the fall in CBF is known as cerebral vascular reserve (CVR), which can be assessed by multiple imaging modalities [3].

Treatment of moyamoya disease revolves around restoring CBF distal to the steno-occlusive disease. Examination of the cerebral vasculature via angiography has long been the gold standard for diagnosis and preoperative surgical planning. Adjunct imaging methods used to measure cerebrovascular hemodynamics such as single photon emission computed tomography (SPECT), positron emission tomography (PET), xenon CT, arterial spin labeling (ASL), magnetic resonance (MR) perfusion, and magnetic resonance angiography (MRA) Non-invasive Optimal Vessel Analysis (NOVA; VasSol, Inc, River Forest, IL) have also been shown to offer advantages when determining management strategies [4]. These metabolic and flow studies allow for pre- and postoperative assessment of disease severity. However, a question of the clinical utility of these various techniques remains.

Most institutions use a variety of modalities, often letting symptomatology ultimately guide treatment. In this study, we evaluated preoperative conventional angiography, MRI, MRA NOVA, as well as pre- and post-acetazolamide challenge SPECT and their impact on the management of patients with moyamoya disease.

## Materials and methods

Institutional protocol

Patients seen at our institution with suspected moyamoya disease are investigated by a standardized institutional protocol. Initial investigations include CT, computed tomography angiography (CTA), or MRA scan depending upon the presenting symptoms. Once moyamoya disease is suspected, patients undergo a brain MRI, cerebral angiography, pre- and post-acetazolamide SPECT scan, and MRA NOVA.

Patients with the symptomatic disease are treated with revascularization when found to have hemodynamic compromise on SPECT with or without acetazolamide, while asymptomatic/mildly symptomatic patients are treated when imaging shows impaired cerebral hemodynamics on SPECT with acetazolamide. Patients with bilateral disease undergo direct and/or indirect bypass on the asymptomatic side if imaging suggests hemodynamic impairment. Direct revascularization is favored in all cases.

Study outline

The Feinstein Institute for Medical Research institutional review board approved a retrospective chart review of patients with moyamoya. Informed consent was waived for this study. All patients seen at our institution with a diagnosis of moyamoya disease were included. Medical records, physicians’ notes, and all radiological studies were reviewed for analysis. Descriptive statistics were used to summarize the data. Fischer's exact test and Mann-Whitney U test were used to calculate statistical differences between datasets.

## Results

Patient characteristics

A total of 34 patients with moyamoya disease were evaluated from January 1, 2011, to January 1, 2016, and received a complete imaging workup as discussed in the methods. The majority of patients were females (76%, 26/34), with an average age of 43 years (range: 17-72; Table 1). Transient or permanent neurological deficit likely due to ischemia was the most common presenting disease process found in 56% of patients (19/34). Three of these patients had transient symptoms (e.g. transient numbness). These three patients had no impaired CVR. Table 2 demonstrates imaging findings categorized by presenting symptoms of the patients.

**Table 1:** Patient demographics

Demographics		
Age	43 (17-72)	
Sex (F)	76% (26/34)	
Side	Left	44% (15/34)
	Right	35% (12/34)
	Bilateral	21% (7/34)
Presentation	Major ischemia	47% (16/34)
	Transient neurological symptoms	9% (3/34)
	Hemorrhage	26% (9/34)
	Headache	6% (2/34)
	Syncope	6% (2/34)
	Seizure	3% (1/34)
	Tinnitus	3% (1/34)

**Table 2:** Imaging findings based on presentation SPECT: single photon emission computed tomography; NOVA: Non-invasive Optimal Vessel Analysis.

		Surgery (% of 26 patients)	No surgery (% of 8 patients)
Presentation	Transient or minor	3 (11.54%)	3 (37.50%)
	Hemorrhage	6 (23.08%)	2 (25.00%)
	Ischemic stroke	17 (65.38%)	3 (37.50%)
MRI findings	Normal	3 (11.54%)	0.00%
	Ischemia	17 (65.38%)	6 (75.00%)
	Hemorrhage	6 (23.08%)	2 (25.00%)
SPECT post Diamox	No change	12 (46.15%)	2 (25.00%)
	Improved	0.00%%	3 (37.50%)
	Decreased	14 (53.85%)	3 (37.50%)
NOVA	Abnormal	26 (100.00%)	8 (100.00%)
	Normal flow	0.00%	0.00%

MRI findings

Brain MRI was abnormal in all but three patients. In total, 65% of patients (22/34) had findings of ischemia (this includes symptomatic and asymptomatic patients), including white matter ischemic disease, or acute infarcts. Of the patients, 26% (9/24) had hemorrhage (Table 1). There was no relationship between MRI findings and the likelihood of a patient undergoing surgery (P > 0.05).

Of the three patients with normal MRI, all patients underwent surgery due to findings of perfusion deficits on SPECT or impaired CVR based on SPECT with acetazolamide. Of the six patients with minor symptoms of moyamoya (headache, transient paresthesia), three were the patients with no significant findings on conventional MRI but impaired hemodynamic imaging. The other three had only radiological findings suggestive of ischemia (Table 2).

Angiographic findings

Findings on conventional cerebral angiography were used as the gold standard for moyamoya disease diagnosis. All patients were found to have evidence of moyamoya on angiography. Suzuki grades ranged from II to VI [2]. In total, 70% of patients presented with Suzuki grade II-IV (Table 1). Only one patient presented with Suzuki grade I.

The six patients with nonclassical symptoms were found to have Suzuki grade III disease in four cases, grade IV in one case, and one with grade V. In three patients (9%), angiography demonstrated a diminutive superficial temporal artery (STA) leading to surgical planning changes and indirect bypass alone.

SPECT method

Post-acetazolamide results were characterized as increased perfusion, decreased perfusion, and no change. In total, 50% (17/34) of patients were found to have decreased perfusion after the addition of acetazolamide and 38% (13/34) had abnormal SPECT with no change after acetazolamide.

In the subcategory of patients with no or minimal symptoms, three patients had normal SPECT before acetazolamide. Another three patients were found to have increased perfusion with the addition of acetazolamide. None of these six patients underwent surgery. Patients who underwent surgery were more likely to have decreased perfusion with the administration of acetazolamide (P < 0.05). There was no significant association between type of presentation (hemorrhage vs. ischemia vs. other) and SPECT findings post acetazolamide (P > 0.05).

MRA NOVA

All patients were found to have abnormal MRA NOVA. For the four patients with normal brain MRI, the MRA NOVA showed decreased flow in the diseased vessels seen on angiography. Flow values were found to be abnormal in all vessels. The average vessel flow on the affected M1 was 13 ml/min, significantly lower than historical controls (P < 0.05) [5].

Treatment

**Figure 1:**
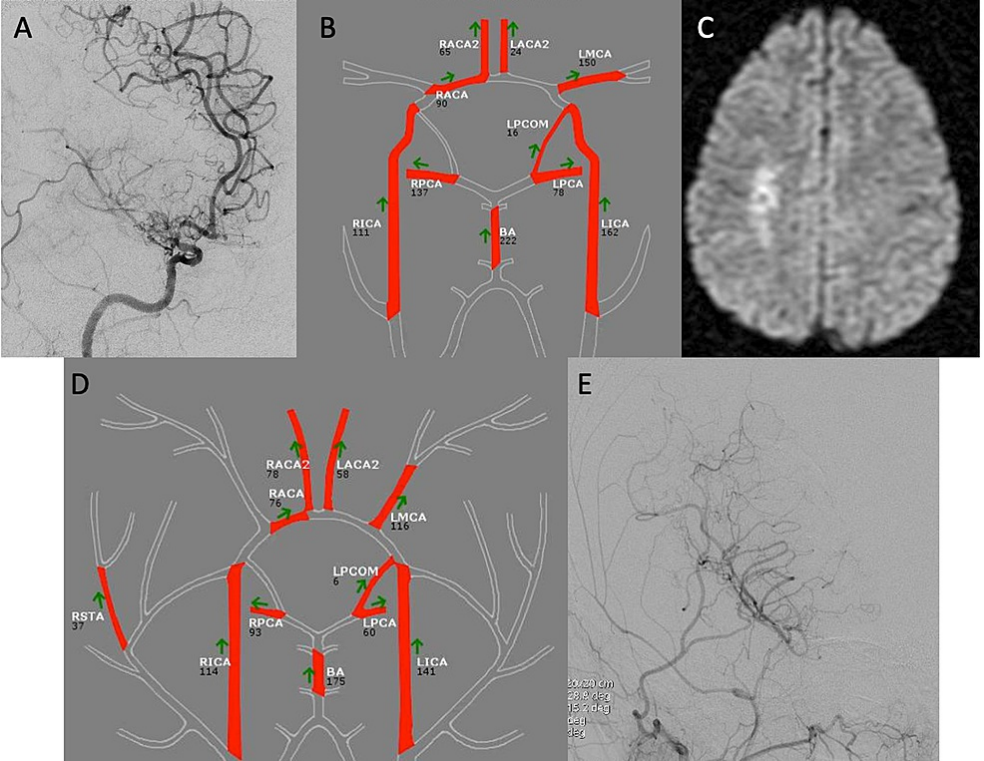
Case example Imaging of an example patient undergoing surgical revascularization. (A) Preoperative angiogram demonstrating supraclinoidal ICA and MCA stenosis as well as early moyamoya collaterals with (B) MRA NOVA demonstrating no flow in the RMCA and (C) showing DWI restriction on initial MRI in a watershed region. The patient underwent right-sided STA to MCA bypass with (D) postoperative MRA NOVA showing bypass flow of 37 ml/min and (E) postoperative angiography of the external carotid artery demonstrating good flow in the bypass graft. ICA: internal carotid artery; MCA: middle cerebral artery; RMCA: right middle cerebral artery; DWI: diffusion-weighted imaging: STA: superficial temporal artery; MRA: magnetic resonance angiography; NOVA: Non-invasive Optimal Vessel Analysis; RACA2: right anterior cerebral artery A2; LACA2: left anterior cerebral artery A2; LMCA: left middle cerebral artery; RACA: right anterior cerebral artery; LPCOM: left posterior communicating artery; RPCA: right posterior cerebral artery; BA: basilar artery; LPCA: left posterior cerebral artery; RICA: right internal carotid artery; LICA: left internal carotid artery; RSTA: right superficial temporal artery.

In total, 26 out of 34 patients underwent 32 surgical bypass procedures. Of the 32 bypasses, 11 were indirect in the form of encephaloduroarteriosynangiosis (EDAS) and the remaining 21 were direct or combined bypasses. Indirect bypass alone was done whenever the donor or recipient vessel size was deemed sub-optimal for a direct bypass and per the surgeon’s discretion. Six patients had follow-up revascularization procedures of their contralateral hemisphere. Eight out of the 34 patients did not undergo surgical revascularization as mentioned above (Table 3). An example case can be seen in Figure 1.

**Table 3:** Patient imaging findings based on surgical decision SPECT: single photon emission computed tomography; MRA: magnetic resonance angiography; NOVA: Non-invasive Optimal Vessel Analysis.

Presentation								
Transient symptoms or minor			Permanent deficit due to ischemia			Permanent deficit due to hemorrhage		
17.6% (9/34)			47% (16/34)			26.4% (9/34)		
MRI			MRI			MRI		
	Negative	33% (3/9)		Negative	0		Negative	0
	Ischemia	56% (6/9)		Ischemia	16/16 (100%)		Ischemia	0
	Hemorrhage	0		Hemorrhage	0		Hemorrhage	100% (9/9)
MRA NOVA			MRA NOVA			MRA NOVA		
	Normal	0		Normal	0		Normal	0
	Abnormal	100% (9/9)		Abnormal	100% (16/16)		Abnormal	100% (9/9)
SPECT with acetazolamide			SPECT with acetazolamide			SPECT with acetazolamide		
	Increased perfusion	33% (3/9)		Increased perfusion	0		Increased perfusion	0
	No change	33% (3/9)		No change	50% (8/16)		No change	22% (2/9)
	Decreased perfusion	33% (3/9)		Decreased perfusion	50% (8/16)		Decreased perfusion	78% (7/9)
Surgery			Surgery			Surgery		
	Yes	33.3% (3/9)		Yes	100% (16/16)		Yes	78% (7/9)
	No	66.7% (6/9)		No	0		No	22% (2/9)

In total, 15% (4/26) of patients experienced complications: one hyperperfusion syndrome, one postoperative stroke, one pseudomeningocele, and one patient with deep vein thrombosis. All complications, except for the postoperative stroke, occurred in patients with direct bypass. In patients undergoing direct bypass, bypass patency was confirmed in all but one patient (20/21 patients, 95% patency). The long-term bypass patency and hemodynamic evaluation are beyond the scope of this paper.

Surgical decision-making and preoperative imaging impact

Surgical decision-making was predominantly influenced by symptomatology for symptomatic patients and by SPECT findings. In total, 76% of patients underwent surgery. Standard MRI failed to change the decision for surgery as the patients with normal MRI all underwent surgery. MRA NOVA was abnormal in all patients, so its impact on decision-making is unclear. Eight patients did not undergo surgery because of their symptomatology or workup. Of those eight, three did not receive surgery as they were minimally symptomatic with normal SPECT findings. Three patients had a SPECT demonstrating increased perfusion (Table 3). Two died prior to surgery due to the initial hemorrhage. However, those two patients would have been surgical candidates had they not passed. Preoperative formal cerebral angiography did alter surgical strategy in three of the minimally symptomatic patients (11%) prompting indirect bypass due to a small STA.

## Discussion

Most patients with moyamoya initially present with symptoms related to their pathology: transient ischemic attack (TIA), infarct, or hemorrhage [1]. Inevitably, a CT or MRI is done. For symptomatic patients, noninvasive vascular imaging is typically obtained with either a CTA or MRA [6,7]. Subsequently, the patient will typically undergo cerebral angiography to confirm the moyamoya diagnosis [6]. At this point, there are currently many diagnostic studies to choose from to aid in the formation of a therapeutic plan. However, we demonstrated in this paper that these modalities may offer little utility in therapeutic decision-making. In the era of rising healthcare costs, efficient and appropriate utilization of resources without jeopardizing outcomes is necessary.

Surgeons and neurologists will obtain hemodynamic imaging studies as monitoring tools to assess the progression of the disease and indication for surgery as well as to assess the efficacy and stability of the surgical intervention [8,9]. In this series, 88.2% of patients had abnormal MRIs. The four patients with normal findings on non-contrast MRI ultimately underwent revascularization. The diagnosis of moyamoya can be reliably made on MRI/MRA alone [7,10]. While MRI may be useful for the diagnosis of moyamoya, it is obviously not sufficient for surgical decision-making.

The patients who did not undergo surgery were (1) those who were determined to be minimally symptomatic without perfusion deficit on hemodynamic imaging, or (2) patients who died prior to surgery. Therefore, all patients with symptoms with anything more than minimal symptoms were treated. Imaging did not change the treatment plan in this patient group because all of these patients invariably had abnormal SPECT and MRA NOVA. Considering the absence of influence of these tests in surgical decision-making, we suggest that the preoperative evaluation of symptomatic moyamoya patients may be limited.

Surgical decision-making was influenced by the results of SPECT with and without acetazolamide in the case of mildly symptomatic patients (i.e. headache or transient sensory deficit). Hemodynamic workup preoperatively in this subset of patients is needed as some of these patients might not benefit from revascularization at the time of presentation. A recent study demonstrated that adult patients with ischemic type moyamoya may be managed conservatively with no revascularization if SPECT fails to show misery perfusion [11].

Aside from surgical decision-making, MRA NOVA and SPECT do establish a quantitative baseline for long-term monitoring. Quantitative MRA can be used to follow bypass flow values and determine long-term bypass patency and recent literature demonstrates that patients with moyamoya have uniformly disturbed intracranial flow [12-14]. One unique value of quantitative MRA is in the postoperative course where it may be used to determine the cause of new neurological deficits and correlate to flow through the bypass. Similarly, one could see the progression of a bypass from lower to higher flow or vice versa to better understand the hemodynamics of a particular bypass [15,16]. Patients undergoing direct bypass are at risk of both stroke and focal deficit from hyperperfusion. Quantitative MRA NOVA can help delineate the cause [17].

Similarly, SPECT can be used to determine the symmetry of perfusion after surgical intervention [17,18]. In cases of bilateral disease, SPECT value can be challenging and requires an accurate assessment of the region of interest, often requiring comparison to the ipsilateral cerebellum [19]. Importantly, a more recent study found that SPECT alone was more sensitive and specific than SPECT with acetazolamide to assess misery perfusion when compared to PET [20]. This is consistent with older literature, which found that SPECT alone correlated highly with findings on PET [21]. Furthermore, another study found that patients without misery perfusion may be treated conservatively with antiplatelet therapy alone, which is contrary to the prior understanding of moyamoya [11,20].

Alternative techniques based on MR imaging including blood oxygen level-dependent MRI have been developed to assess hemodynamic parameters [22]. Of note, ASL has demonstrated some utility in patients with moyamoya disease. These techniques, using a single modality, allow for the assessment of CBF, perfusion parameters, and diagnosis of moyamoya disease [23-25]. Most likely as MR continues to advance, all hemodynamic measures will be obtained through single modality imaging and multimodality tests may be phased out. Conventional angiography, however, will remain the gold standard diagnostic test. Ultimately, this study demonstrates that while multimodal investigations may aid in treatment planning, the decision to treat symptomatic moyamoya patients can be made using limited information. We provide evidence that symptomatic moyamoya patients can undergo treatment after angiographic confirmation without any preoperative hemodynamic testing. While hemodynamic imaging studies can aid in establishing a preoperative baseline of CBF and CVR for follow-up studies, the actual implication of these tests in the preoperative evaluation of symptomatic moyamoya patients is debatable. For symptomatic patients, preoperative hemodynamic testing may be unnecessary as revascularization is generally indicated. For asymptomatic/mildly symptomatic patients or those presenting with nonclassical symptoms; however, hemodynamic studies are necessary to determine the need for treatment.

There are significant limitations of this study including its retrospective nature, small sample size, and lack of significant follow-up, especially for minimally symptomatic patients. Another significant limitation of this study is the lack of hemodynamic imaging follow-up. Our conclusions regarding surgical decision-making as it relates to imaging results largely reflect the clinical judgment of the authors. We believe, however, that these clinical decisions are generalizable to the broader neurosurgical and neurological communities.

## Conclusions

In our cohort of patients with moyamoya disease and syndrome, it was initial presenting symptoms that drove the decision for surgery. Advanced hemodynamic imaging was useful as a noninvasive tool to track surgery to evaluate patients with only minor symptomatology and to assess contralateral disease burden.
